# Disruption of type 3 adenylyl cyclase expression in the hypothalamus leads to obesity

**DOI:** 10.15761/IOD.1000149

**Published:** 2016-05-13

**Authors:** Hong Cao, Xuanmao Chen, Yimei Yang, Daniel R Storm

**Affiliations:** 1 Institute of Neurobiology, Institutes of Brain Science and State Key Laboratory of Medical Neurobiology, Fudan University, China; 2 Department of Pharmacology, School of Medicine, University of Washington, USA; 3 Department of Molecular, Cellular, and Biomedical Sciences, University of New Hampshire, USA

**Keywords:** type 3 adenylyl cyclase, hypothalamus, obesity

## Abstract

Evidence from human studies and transgenic mice lacking the type 3 adenylyl cyclase (AC3) indicates that AC3 plays a role in the regulation of body weight. It is unknown in which brain region AC3 exerts such an effect. We examined the role of AC3 in the hypothalamus for body weight control using a floxed AC3 mouse strain. Here, we report that AC3 flox/flox mice became obese after the administration of AAV-CRE-GFP into the hypothalamus. Both male and female AC3 floxed mice showed heavier body weight than AAV-GFP injected control mice. Furthermore, mice with selective ablation of AC3 expression in the ventromedial hypothalamus also showed increased body weight and food consumption. Our results indicated that AC3 in the hypothalamus regulates energy balance.

## Introduction

Obesity is a major health issue associated with complications that cause significant morbidity and mortality. Obese individuals have a higher risk for a number of diseases including type 2 diabetes, cardiovascular disease, metabolic syndrome, hypertension, certain forms of cancer, and sleep-breathing disorders [1]. Furthermore, obesity decreases longevity and lowers the general quality of life [2–5]. Although intensive effort has been devoted to anti-obesity therapy, the percentage of obese individuals in industrialized countries continues to increase.

Adenylyl cyclases (ACs) catalyze the synthesis of cyclic 3′5′-AMP (cAMP) from ATP. There are ten AC isoforms that have been cloned and characterized in mammals [6]. It has been reported that AC3 gene polymorphisms are associated with obesity in a group of Swedish men [7]. In another study, a genome-wide association analysis based on height-adjusted BMI found that SNPs in AC3 were associated at genome-wide significance level (rs11676272 (0.28 kg/m3.1 change per allele G (0.19, 0.38), P56 3 1029). The association signal at AC3 is apparently driven by a miss-sense variant [8]. These human genetic studies suggested that AC3 might play an important role in the regulation of body weight. Previous studies from our lab showed that a global AC3 mouse knockout exhibit adult onset obesity [9]. They show increased fat mass, larger adipocytes, reduced physical activity, increased food consumption, and leptin insensitivity. Subsequently, it was discovered that a gain of function mutation in AC3 protects mice from diet-induced obesity providing further evidence that AC3 may play a major role in weight control [10]. However, why AC3^−/−^ mice are obese remains unclear.

AC3 is expressed throughout the nervous system and AC3^−/−^ mice exhibit a number of other phenotypes including anosmia, depression, and defects in extinction of hippocampus dependent memory that confound interpretation of the obesity phenotype in the global AC3 knockout. The importance of the hypothalamus in regulating energy balance has been well established [11–13]. For example, ablation of the ventromedial nuclei of the hypothalamus causes overeating and obesity [14]. Since AC3 is expressed in several tissues and various areas of the brain including the hypothalmus, the goal of this project was to determine if AC3 ablation in the hypothalamus causes adult onset obesity using a floxed AC3 mouse strain.

## Methods

### Mice

Mice were housed at 22–24°C with a 12-h light/dark cycle, and had access to food and water *ad libitum*. The age of animals used in this study was 2 – 4 months old. AC3 lox/lox mice were generated as previously reported [15]. All animal procedures were approved by the Institutional Animal Care and Use Committee at the University of Washington and performed in accordance with their guidelines.

### Body weight and food consumption

Body weight was monitored before virus injection, then weekly after injection. Food intake was measured daily on the fourth week after virus injection.

### Immunofluorescene

Immunofluorescene procedures were performed as described previously with a few modifications [9]. Mice were perfused with 4% paraformaldehyde (PFA). The brains were then dissected and postfixed with the same fixing solution overnight at 4°C. Then the brains were cryoprotected with 30% sucrose for 48 hr at 4°C. Brains were cut into 30 mm sections with a cryostat freezing microtome. Floating sections were blocked and then first incubated with the AC 3 antibody (1:500, Santa Cruz Biotechnology, Inc.) overnight at 4°C followed by the secondary antibody, Alexa Fluor 488-conjugated donkey anti-rabbit IgG (Invitrogen, La Jolla, CA), for 2 hr at room temperature. Stained sections were mounted onto microscopy slides and visualized by confocal microscopy (Zeiss 510 META).

### Stereotaxic viral injection

AAV1-Cre-GFP and AAV1- GFP were bilaterally injected into the hypothalamus (2.2 × 10^11^ viral genome per microliter) of 4-wk-old AC3 lox/lox mice. According to previous report [16], four-site injections of 0.5 μL per site were performed for each side at the coordinates x = ± 0.5, y = −1.4, and z = −5.6 and −5.0, which corresponds to ventral and dorsal hypothalamus, respectively. For the VMH injection, two-site injections were performed for each side at the coordinates x = ± 0.5, y = −1.4, and z = −5.6. GFP fluorescence was used to identify the virally infected areas. AC3 immunohistochemistry was used to show the expression of AC3. Body weight was monitored before and after virus injection for each mouse. Results from animals that received injections at the correct site as determined by GFP fluorescence were included in analyses.

### Statistical analysis

All data are expressed as means ± SEM. Data from food consumption were analyzed using the unpaired Student’s t test. For body weight curves, the data were analyzed with two-way ANOVA followed by Holm-Sidak Test. P<0.05 was considered to be statistically significant.

## Results

**Figure 1:**
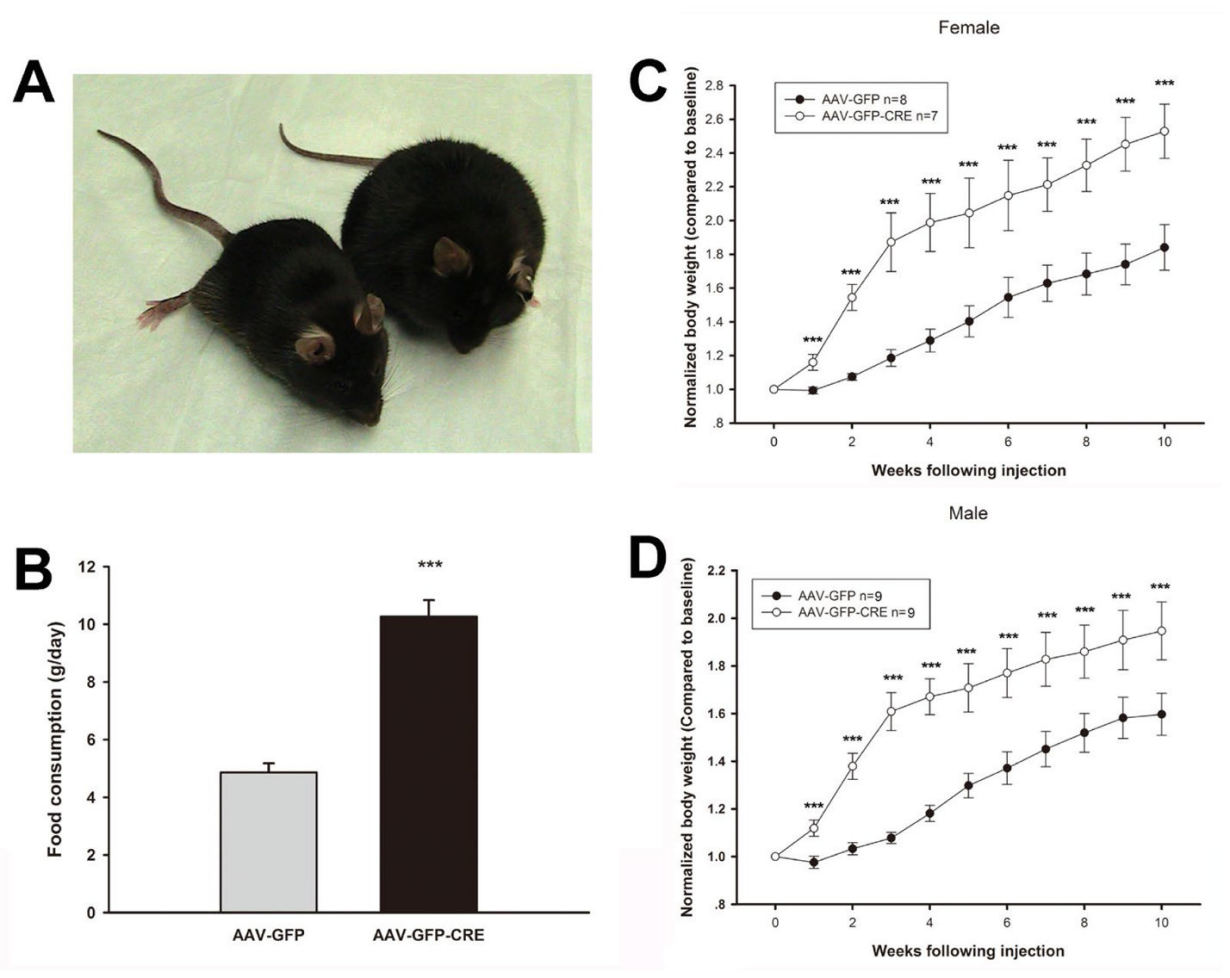
AC3 in the hypothalamus contributes to obesity. A. Representative mice at weeks 10 after AAV-GFP and AAV-GFP-CRE injections. B. Daily food consumption of AC3 lox/lox mice with AAV-GFP and AAV-GFP-CRE injections. C-D. Body weight in female (C), Male (D) mice after introhypothalamus injection of AAV-GFP and AAV-GFP-CRE.

**Figure 2:**
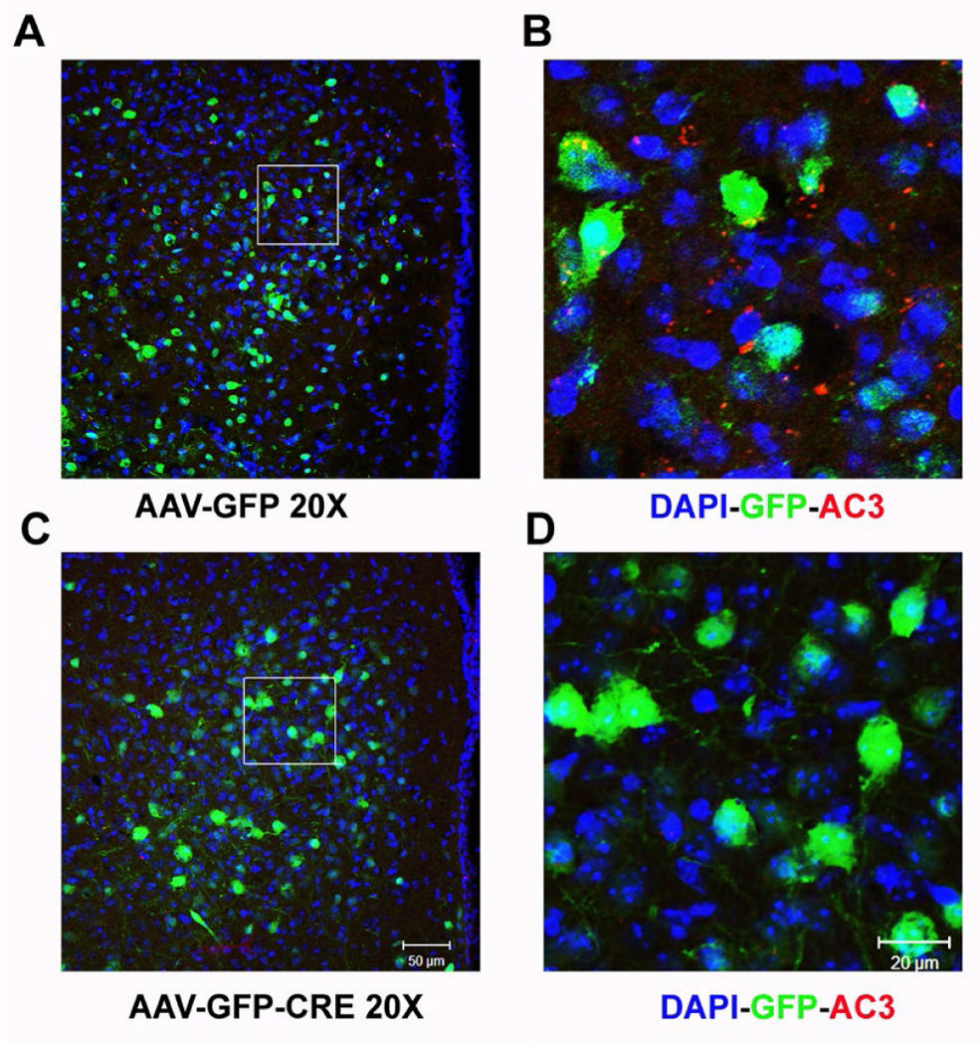
The immunohisto chemistry showing AC3 (red) expression in the hypothalamus following AAV-GFP injection (A and B) and AAV-GFP-CRE injections (C and D), DAPI (blue), GFP (green) and AC3 (red). Injection site: AP −1.46 mm posterior to Bregma; ML ±0.5 mm; DV −5.0 & −5.5 mm.

To investigate the role of AC3 in the hypothalamus for regulation of body weight, we injected a recombinant virus AAV-CRE-GFP bilaterally into the hypothalamus of AC3 lox/lox mice, which deletes AC3 expression specifically in the hypothalamus. As showed in Figure 1, AAV-CRE-GFP injected mice showed an obvious weight gain compared to AAV-GFP injected mice 10 weeks later. Both male (Figure 1C) and female (Figure 1D) mice that received the AAV1-CRE-GFP construct were significantly heavier than floxed AC3 mice that received an AAV-GFP construct as a control during the period examined (Male, Two way ANOVA, F_(1, 170)_ = 42.67, P < 0.001 N=9 for both groups; Female, Two way ANOVA, F_(1, 137)_ = 54.6123, P < 0.001; N=8 for GFP control group and N=7 for CRE-GFP group). Adult male floxed AC3 with AAV-CRE-GFP injection are about 20% heavier that the floxed mice with AAV-GFP injection, while female floxed AC3 mice are 40% heavier. In addition, the AC3 flox/flox mice with AAV1-CRE-GFP injection consumed twice as much food daily than the control mice, suggesting that increased food consumption may contribute to the obesity of AC3 floxed mice with AAV-CRE injection. (CRE-GFP *vs.* GFP (g): 10.26 ± 0.58 *vs* 4.86 ± 0.31, P < 0.001)(Figure 1B). We verified that AC3 expression in the hypothalamus was reduced when AAV1-CRE-GFP was injected into the hypothalamus of floxed AC3 mice using a ployclonal antibody against AC3 (Figure 2).

**Figure 3:**
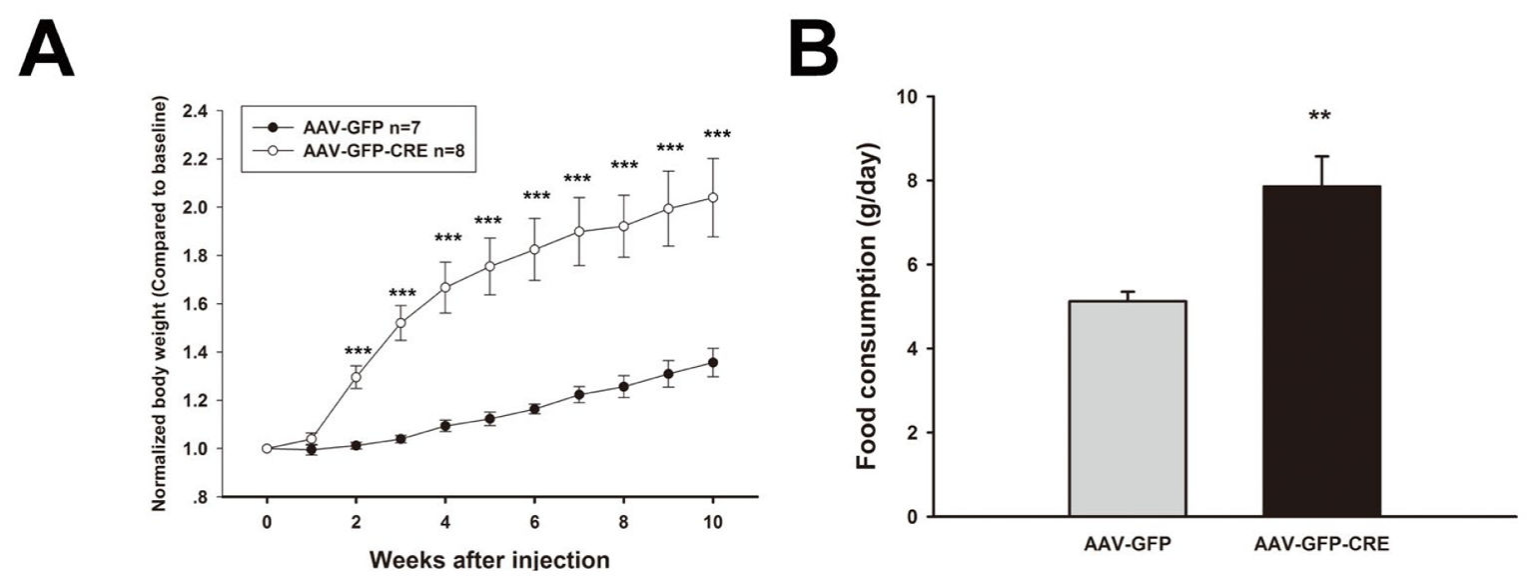
The mice with specific ventromedial hypothalamus injection of AAV-GFP-CRE develop obesity. A. The body weight of AC3 lox/lox mice with VMH injection of AAV-GFP and AAV-GFP-CRE respectively. B. Daily food consumption of AC3 lox/lox mice after AAV-GFP and AAV-GFP-CRE injections.

Several different sites within the hypothalamus are known to be important in regulating body weight including the ventromedial hypothalamic nucleus (VMH). For example, bilateral lesions of the VMH in rats produced a severe hyperphagic obesity syndrome. Therefore, we selectively disrupted AC3 expression in the VMH by injecting AAV1-CRE-GFP to explore whether loss of AC3 in the VMH contributes to obesity (Figure 3). Mice with AAV1-CRE-GFP VMH injection exhibited significant heavier body weight (Two way ANOVA, F_(1, 141)_=73.017, P < 0.001, N=7 for control group and N=8 for AAV-CRE-GFP injected group), higher food consumption (CRE-GFP vs GFP, 7.856 ± 0.718 vs 5.126 ± 0.257, P =0.005).

## Discussion

The hypothalamus plays a central role in maintaining energy homeostasis. Different hypothalamic nuclei, including the arcuate nucleus (ARC), dorsomedial hypothalamus (DMH), ventromedial hypothalamus (VMH), paraventricular nucleus (PVN), and lateral hypothalamic area (LH) have been demonstrated to have specific functions in the regulation of energy balance [11–13]. Earlier evidence has reported that lesions in the VMH produced robust increases in adiposity, caused obesity, and in some strains, diabetes [14,17]. In the present study, we found proved that selective ablation of AC3 in the hypothalamus caused obesity. Interestingly, selective ablation of AC3 in the hypothalamus VMH lead to obesity.

It has been reported that AC3 gene polymorphisms are associated with obesity in humans [7,8]. We previously reported that disruption of the AC3 gene in mice cause obesity as they aged. Adult male AC3^−/−^ mice are about 40% heavier than wild type male littermates while female AC3^−/−^ mice are 70% heavier. The gain in weight is due to an increase in body fat. Although lipolysis is unaltered in AC3^−/−^ mice, their additional weight is due to leptin insensitivity, increased fat mass and larger adipocytes. Before the onset of obesity, young AC3^−/−^ mice (2 months) exhibit reduced physical activity, increased food consumption, and leptin insensitivity [9]. This suggests that the obesity of AC3^−/−^ may be due to loss of AC3 in the hypothalamus. To address this issue we made a floxed AC3 mouse for the selective disruption of AC3 expression in various areas of the CNS [15].

Selective ablation AC3 expression in AC3 flox/flox mice using VMH-injection of AAV-CRE-GFP significantly increased the body weight, supporting the hypothesis that AC3 in the ventromedial hypothalamus plays an important role in control of weight. A possible contributor to obesity caused by AC3 ablation is leptin insensitivity. Leptin can act in the hypothalamus to reduce food intake and increase physical activity [18]. The leptin receptor is a JAK-STAT receptor and it is not directly coupled to adenylyl cyclase activity. The role of AC3 in leptin insensitivity is therefore likely to be downstream of the leptin receptors in the ventromedial hypothalamus. It has been reported that the VMH mediates the leptin-induced increase in glucose utilization as well as its insulin sensitivity in the whole body through activation of the melanocortin receptor (MCR). Leptin sensitivity is due, in part, to production of alpha-MSH which stimulates adenylyl cyclase activity in MC4-R-expressing neurons in the ventromedial hypothalamus [19]. POMC neurons originating in the arcuate nucleus contact synaptically at MC4-R containing neurons in the VMH or other hypothalamic nuclei that participate in control of feeding behavior [19]. The importance of MC4-Rs in control of body weight is illustrated by the presence of extreme obesity in MC4-R gene knockout mice and in humans with mutations in MC4-R [20,21]. Furthermore, the injection of agonists of the MC4-R ICV into mice significantly depresses food consumption while chronic blockade of MC4-R causes obesity [22]. Since AC3 is one of the major adenylyl cyclases expressed in the hypothalamus and AC3^−/−^ mice exhibit obesity, it is possible that MC4-R receptors normally couple to stimulation of AC3 in neurons of the VMH to generate cAMP signals which lead to appetite suppression and/or energy utilization. Hence, the obesity of AC3 flox/flox mice after AAV-CRE-GFP administration may be due to loss of cAMP production in VMH neurons in response to melanocortins, thereby leading to obesity. Taken together, our results indicated that AC3 might be a potential drug target site to combat obesity.
